# Synthesis, Characterization and In Vitro Evaluation of a Novel Glycol Chitosan-EDTA Conjugate to Inhibit Aminopeptidase-Mediated Degradation of Thymopoietin Oligopeptides

**DOI:** 10.3390/molecules22081253

**Published:** 2017-07-26

**Authors:** Jiao Feng, Yan Chen, Feng Li, Lili Cui, Nianqiu Shi, Wei Kong, Yong Zhang

**Affiliations:** 1 National Engineering Laboratory for AIDS Vaccine, School of Life Sciences, Jilin University, Changchun 130012, China; fengjiao0702@gmail.com (J.F.); chen_yan@jlu.edu.cn (Y.C.); fengli15@mails.jlu.edu.cn (F.L.); weikong@jlu.edu.cn (W.K.); 2 Key Laboratory for Molecular Enzymology and Engineering the Ministry of Education, School of Life Sciences, Jilin University, Changchun 130012, China; shinianqiu2009@163.com; 3 Institute of Pharmaceutical Science, King’s college London, Franklin-Wilkins Building, 150 Stamford Street, London SE1 9NH, UK; lilicuinike@gmail.com; 4 Department of Pharmaceutics, School of Pharmacy, Jilin Medical University, Jilin 132013, China

**Keywords:** chitosan, peptide degradation, chelation, conjugation

## Abstract

In this study, a novel conjugate consisting of glycol chitosan (GCS) and ethylene diamine tetraacetic acid (EDTA) was synthesized and characterized in terms of conjugation and heavy metal ion chelating capacity. Moreover, its potential application as a metalloenzyme inhibitor was evaluated with three thymopoietin oligopeptides in the presence of leucine aminopeptidase. The results from FTIR and NMR spectra revealed that the covalent attachment of EDTA to GCS was achieved by the formation of amide bonds between the carboxylic acid group of EDTA and amino groups of GCS. The conjugated EDTA lost part of its chelating capacity to cobalt ions compared with free EDTA as evidenced by the results of cobalt ion chelation-mediated fluorescence recovery of calcein. However, further investigation confirmed that GCS-EDTA at low concentrations significantly inhibited leucine aminopeptidase-mediated degradation of all thymopoietin oligopeptides.

## 1. Introduction

**Figure 1:**
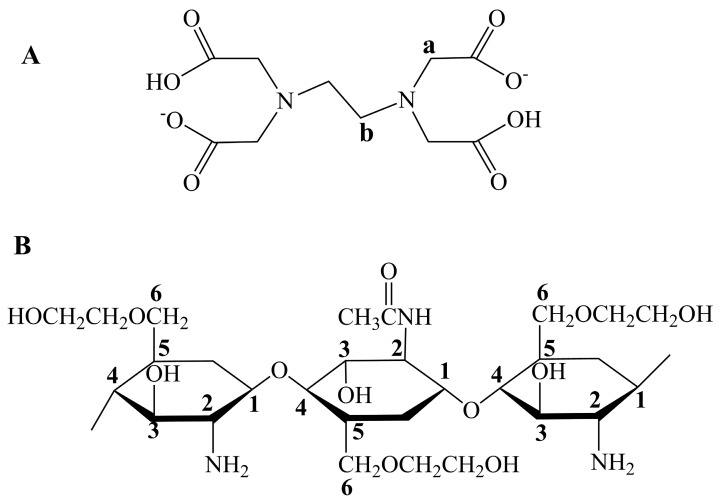
Structures of EDTA (**A**) and glycol chitosan (GCS) (**B**). The protons along the backbones of EDTA and GCS are numbered for subsequent peak assignments in NMR spectra.

Glycol chitosan is one of the polysaccharides that are soluble at pH values from 0 to 14, while conventional chitosan cannot disperse at a monomolecular level at pH values higher than 6.5 [1,2]. The significant difference originates from the replacement of hydrogen by ethylene glycol residues at the hydroxyl group bonded to the 6th C of the glucosamine ring of chitosan (Figure 1). The solubility property gives glycol chitosan great advantages over chitosan in conjugation modifications, especially for the reaction conditions required at neutral or alkali pH values. Furthermore, the resultant conjugates are usually soluble at all pH values due to the intrinsic solubility property of glycol chitosan [3]. In addition, similar to chitosan, glycol chitosan is biodegradable and biocompatible. Recently, glycol chitosan has been proved to have the ability to stabilize lipid rafts in the intestinal brush border and to inhibit the P-glycoprotein efflux pump through membrane binding [4,5].

Covalent attachment of functional molecules, such as enzyme inhibitors and thiol bearing ligands, to chitosan has been widely reported [6]. The main purpose is to endow chitosan with other functions, such as enzyme inhibition and stronger mucoadhesive property. However, few studies involve glycol chitosan and its modification mainly focuses on hydrophobic ligand conjugation to yield amphiphilic polymers capable of self-assembling in aqueous solutions [7,8,9]. In our previous studies, we reported a novel glycol chitosan-bestatin conjugate, and evaluated its potential application as an aminopeptidase inhibitor to protect thymopoietin oligopeptides from enzymatic degradation [3]. Aminopeptidase, as zinc ion-dependent metalloenzyme, is widely distributed in nasal mucosa and the brush-border membrane of the gastrointestinal tract and is the key enzyme to rapidly degrade thymopoietin oligopeptides via nonparenteral administration routes [10,11,12]. Inhibition of aminopeptidase can be achieved through competitive binding of the active site of the enzyme or through chelation-mediated removal of zinc ions from its active site. Glycol chitosan-bestatin is an effective inhibitor as free bestatin through enzyme-substrate binding interaction. However, highly efficient inhibition can only be achieved in the presence of a high concentration of conjugates [3]. Specific inhibition may be less advantageous in protecting peptides and proteins from extensive degradation by other enzymes via the nonparenteral administration routes, such as nasal mucosal and oral delivery.

EDTA is an efficient broad-spectrum inhibitor of metalloproteases via the metal ion-chelating mechanism, and usually requires low concentration such as 1–10 µM. However, EDTA with free carboxyl groups is barely soluble and often requires higher pH than 7 to achieve monomolecular dispersion, where two of four carboxyl groups will be ionized [13]. In addition, when used as an enzyme inhibitor for oral delivery of proteins and peptides, high dose is generally needed because of extensive dilution and clearance during passage. In this case, the safety problem may arise especially for the long-term usage due to the possible interference to metal ion-dependent biological processes [14]. Conjugation of EDTA to chitosan can partly solve the above problems through mucoadhesion. However, the synthesis reaction requires complicated pH adjustment, and the resultant conjugate is insoluble in acid medium due to the attachment of EDTA to primary amino groups of chitosan [15]. In addition, the conjugate usually forms transparent gel, but not real solution, in neutral and alkali solutions [15,16]. Considering the intrinsic solubility property of glycol chitosan, using glycol chitosan as conjugation matrices will probably have more advantages, such as yielding a solution at all pH values. In this study, glycol chitosan-EDTA was synthesized by forming amide bonds between the amino group of the latter and the carboxylic acid group of the former. The influence of conjugation on the chelation capacity of EDTA to cobalt ions was evaluated. Moreover, its potential application as a metalloenzyme inhibitor in solution was investigated using three thymopoietin oligopeptides as model peptide drugs in the presence of leucine aminopeptidase.

## 2. Results and Discussion

### 2.1. FTIR Measurement

**Figure 2:**
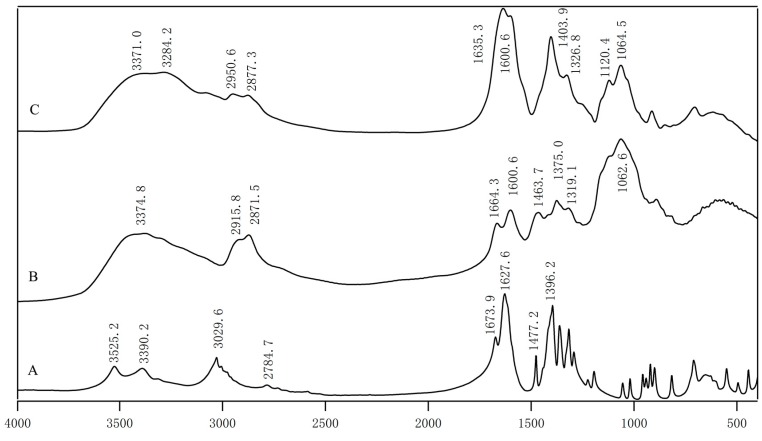
The FTIR spectra of Na_2_-EDTA (**A**); GCS (**B**); GCS-EDTA (**C**) synthesized with a GCS:EDTA mass ratio of 1:30 and followed by complete removal of free EDTA.

The FTIR spectra of EDTA-Na_2_, glycol chitosan (GCS) and GCS-EDTA are shown in Figure 2. Among the spectra, EDTA-Na_2_ gave three typical absorptions of carboxylic groups at 1673.9, 1627.6 and 1396.2 cm^−1^, respectively. The results agreed very well with previous reports [17,18]. The peak assignment of glycol chitosan is as follows. The broad peak at 3374.8 cm^−1^ comes from amine N-H stretch, which is overlapped with O-H stretch vibration [19]. Peaks at 2915.8 and 2871.5 cm^−1^ are the typical C-H stretch vibration. Two peaks centered at 1664.3 and 1600.6 cm^−1^ represented amide I and II respectively [16]. In the GCS-EDTA spectrum, the peaks at 1673.9 and 1664.3 cm^−1^ disappeared and a new band appeared at 1635.3 cm^−1^, indicating the formation of amide groups, although the typical absorption of carboxylic groups at 1627.6 cm^−1^ might overlap with amide I. Another new strong band centered at 1403.9 cm^−1^, which is characteristic of carboxylic groups of EDTA, further confirmed the successful attachment of EDTA to glycol chitosan. It should be noted that, before FTIR measurement, GCS and GCS-EDTA experienced extensive dialysis treatment, and therefore the peaks in FTIR spectra of GCS-EDTA can only originate from the conjugate but not from the free EDTA. In addition, considering the excessive EDTA used in the conjugation reaction, one mole free amino group is expected to conjugate with one mole EDTA group.

### 2.2. NMR Measurement

**Figure 3:**
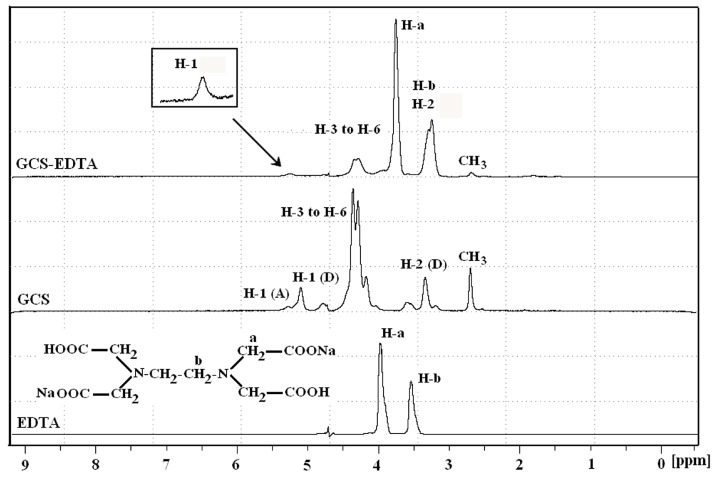
^1^H-NMR spectra of Na_2_-EDTA, GCS and GCS-EDTA synthesized with a GCS:EDTA mass ratio of 1:30 in pOD 10 solutions at 70 °C. The numbered protons along the backbones of EDTA and GCS are shown in Figure 1. The (D) refers to deacetylated residues, while the (A) refers to acetylated residues.

To further confirm the successful conjugation, NMR was used to characterize the resultant conjugates in an alkali solution. The ^1^H-NMR spectra of EDTA-Na_2_, unmodified and EDTA-modified GCS are shown in Figure 3. In the case of EDTA-Na_2_, peaks at 3.97 and 3.53 ppm resulted from protons H-a and H-b on the methylene groups of EDTA, respectively, which agreed well with the ratio of relative integrals. For GCS, the peaks assigned to the protons along the backbones of GCS are numbered in Figure 1B. The proton assignments of GCS were: δ 2.69 (3H, CH3 of the acetyl group), δ 3.34 (1H, H-2 (D)), δ 4.00–4.60 (5H, H-3, 4, 5, and 6 of glucosamine ring), δ 5.09 (1H, H-1 (D)) and δ 5.28 (1H, H-1 (A)) [20,21]. The degree of acetylation (DD) calculated by the equation of DD = (I_CH3_/3)/(I_H-1total_) was 26.08% [16]. In contrast with GCS, GCS-EDTA showed characteristic proton signals in the range 3.0–4.0 ppm. The peak centered at 3.75 ppm was the proton signal of H-a on the methylene groups of EDTA, while the peak at around 3.26 ppm was the overlap of those of H-2 of GCS and H-b on the methylene groups of EDTA. The peaks arising at 3.75 ppm and 3.26 ppm indicated the successful conjugation of GCS with EDTA as GCS-EDTA experienced extensive dialysis treatment before NMR measurement. In addition, the peak resulted from H-1 (D) of GCS disappeared in the spectra of GCS-EDTA, suggesting that there was no free amino group present in this molecule. The calculated degree of acetylation was 26.71%, in agreement with that of free GCS, suggesting that conjugation reaction did not have a remarkable effect on DD. Together with the results of FTIR, it is reasonable to conclude that the conjugation of EDTA to glycol chitosan is successfully achieved.

### 2.3. Evaluation of Chelating Ability

**Figure 4:**
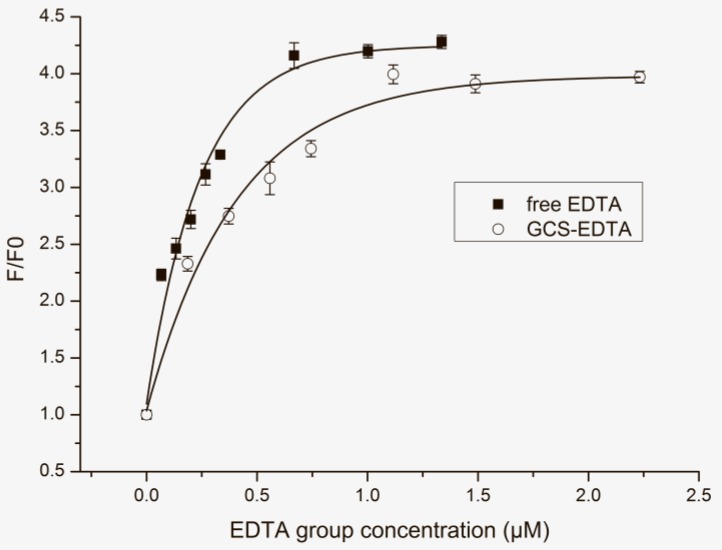
The effect of cobalt ion chelation resulted from EDTA and GCS-EDTA with different EDTA group concentrations on the normalized maximum fluorescence emission intensity of calcein (0.0625 µg/mL). The molar ratio of calcein to cobalt dichloride was 1:1. Data were fitted with the Asymptotic Regression Model in software OriginLab of version 8.5 (OriginLab, Northampton, MA, USA), and adjusted R^2^ EDTA and GCS-EDTA were 0.97 and 0.98, respectively.

Whether the successful conjugation will influence the chelation capacity of EDTA is a key point to its future application. Figure 4 shows the effect of EDTA and GCS-EDTA at different concentrations on the normalized fluorescence intensity of calcein in the presence of cobalt ions. It should be noted that molar concentrations of the EDTA group were used for both EDTA and GCS-EDTA. For the latter, the EDTA molar concentration was calculated according to the speculation that all free amino groups of glycol chitosan were conjugated with EDTA at 1:1 molar ratio. The reasonable speculation is supported by the results of FTIR and NMR assays and other studies [15]. As shown in Figure 4, with the increasing concentration of EDTA, the fluorescence intensity of calcein gradually increased, suggesting concentration-dependent chelation of EDTA to cobalt ions. When EDTA concentration was higher than 0.67 µM, a plateau was reached, indicating the almost complete removal of cobalt ions from the calcein/cobalt ion complex. In contrast, similar tendency was also found in the presence of GCS-EDTA. However, compared with free EDTA, GCS-EDTA in all molar concentration ranges showed slightly reduced chelation capacity to cobalt ions, which is probably due to the loss of one of four carboxyl groups that resulted from the conjugation. The reasonable speculation is supported by the fact that the chelation of EDTA to metal ions usually involves its two amines and four carboxyl groups.

### 2.4. Inhibition of Aminopeptidase-Mediated Peptide Degradation

**Figure 5:**
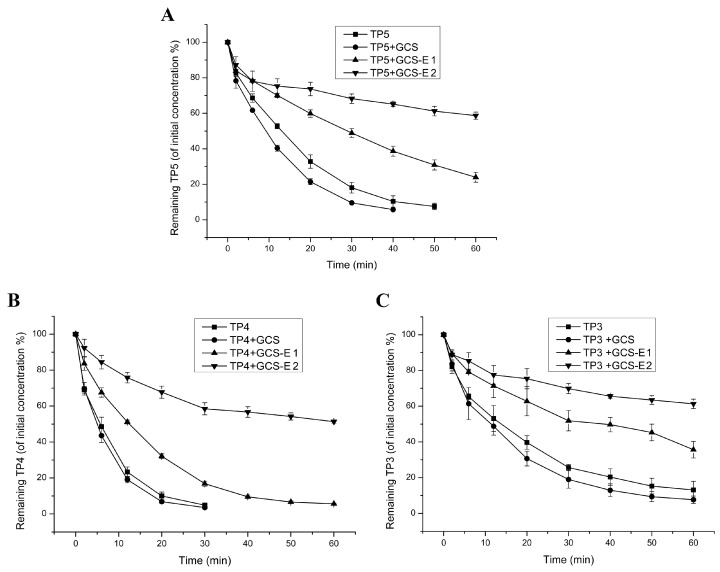
Degradation kinetics of TP5 (**A**), TP4 (**B**) and TP3 (**C**) in the absence and presence of GCS and GCS-EDTA. In all groups, each peptide (0.1 mM) was incubated with LAP (0.01 U/mL) at 37 °C. GCS, 0.50% (*w*/*v*); the concentrations of GCS-EDTA synthesized with a GCS:EDTA mass ratio of 1:30 in group GCS-E 1 and 2 were 0.0001 mg/mL and 0.01 mg/mL, respectively.

**Figure 6:**
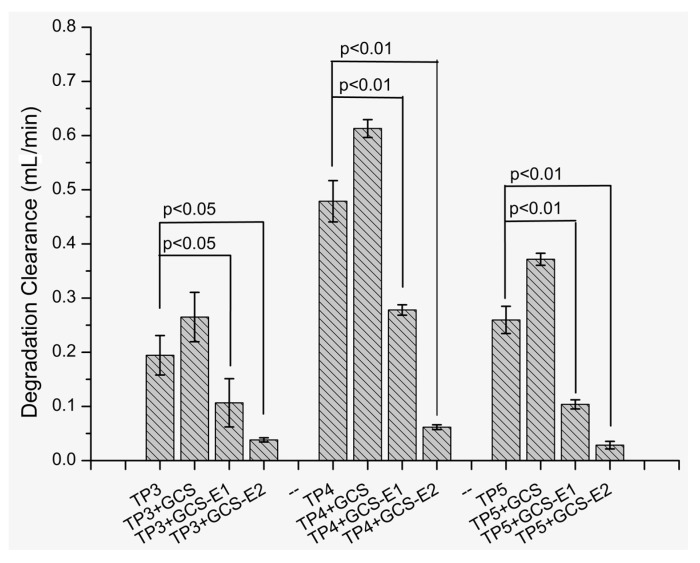
Degradation clearance of TP3, TP4 and TP5 in the absence and presence of GCS and GCS-EDTA. In all groups, each thymopoietin oligopeptide (0.1 mM) was incubated with LAP (0.01 U/mL) at 37 °C. GCS, 0.50% (*w*/*v*); the concentrations of GCS-EDTA synthesized with a GCS:EDTA mass ratio of 1:30 in group GCS-E 1 and 2 were 0.0001 mg/mL and 0.01 mg/mL, respectively.

Here, we investigated the potential application of GCS-EDTA to inhibit aminopeptidase-mediated peptide degradation. The results were shown in Figure 5A–C for TP5, TP4 and TP3, respectively. In the case of TP5, leucine aminopeptidase at 0.01 U/mL caused significant degradation as evidenced by the decreased concentration of TP5 over time. In addition, the addition of GCS slightly accelerated TP5 degradation, which might be due to the residual metal ions-mediated enzyme activation from GCS. Similar phenomena were also observed in our previous studies [3]. However, in the presence of GCS-EDTA, TP5 degradation that resulted from leucine aminopeptidase was significantly inhibited and, at almost all sampling points, the remaining TP5 possessed higher concentrations than those without GCS-EDTA. For TP4 and TP3, similar phenomena were also observed. These results indicated that GCS-EDTA can markedly inhibit the activity of leucine aminopeptidase. However, unexpectedly, 100-time improvement in GCS-EDTA concentration did not completely inhibit aminopeptidase-mediated thymopoietin oligopeptide degradation. Leucine aminopeptidase has been reported to include two zinc ions at its active site in the C-terminal domain [22,23]. One of them is responsible for forming a stable complex with the substrate and another is to activate the catalytic water located in the active site. Removal of one of them may markedly influence its activity and removing all of them will lead to complete inactivation [22]. The incomplete GCS-EDTA inhibition of leucine aminopeptidase may be due to the dynamic equilibrium of zinc ions between the GCS-EDTA/zinc ion complex and enzyme/zinc ion complex. To further reveal GCS-EDTA inhibition of leucine aminopeptidase, the degradation clearance of three peptides in the absence and presence of the conjugate were calculated, and the result is shown in Figure 6. Degradation clearance data agreed well with those of degradation kinetics discussed previously. Interestingly, at the same conditions, three peptides showed different levels of susceptibility to leucine aminopeptidase and gave the order of TP4 > TP5 > TP3. The results are in agreement with several previously published reports [3,24], and can be explained by their different binding energies to aminopeptidase [3]. The findings suggested that combining GCS-EDTA and peptides (such as TP4 and TP3) with robust resistance against enzymatic degradation might achieve an efficient delivery via mucosal routes.

### 2.5. Cytotoxicity of GCS-EDTA

**Figure 7:**
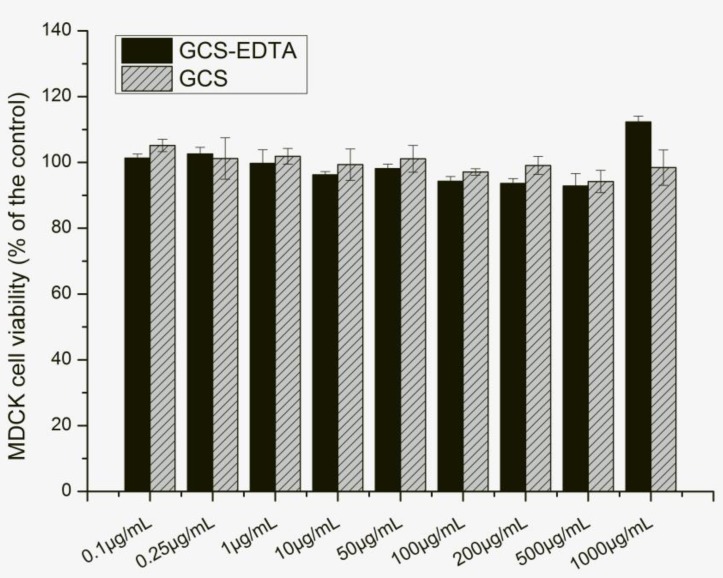
Effect of GCS and GCS-EDTA on the viability of Madin-Darby Canine Kidney (MDCK) cells.

The cytotoxicity of GCS-EDTA and GSC was evaluated by 3-(4,5-dimethylthiazol-2-yl)-2,5-diphenyltetrazolium bromide (MTT) assay after 24-h incubation with Madin-Darby Canine Kidney (MDCK) cells. The results were shown in Figure 7. Both GCS and GCS-EDTA showed dose-dependent negative effects on MDCK cell viability, with the latter resulting in a slightly lower level of cell viability. However, even at all concentrations used in this study, cell viabilities of more than 93% were obtained with all tested samples of GCS-EDTA, suggesting an acceptable level of biocompatibility.

## 3. Materials and Methods

### 3.1. Materials

Thymotrinan (Arg-Lys-Asp, TP3), thymocartin (Arg-Lys-Asp-Val, TP4), thymopentin (Arg-Lys-Asp-Val-Tyr, TP5) and their metabolites were provided by GL Biochem Ltd. (Shanghai, China). All peptide purities were higher than 98% as evidenced by RP-HPLC assay (Agilent Technologies, Santa Clara, CA, USA). Calcein, cobalt hydrodichloride, *N*-hydroxysuccinimide (NHS), leucine aminopeptidase (EC 3.4.11.2, microsomal from porcine kidney), *N*-(3-dimethylaminopropyl)-*N*′-ethylcarbodiimide hydrochloride (EDAC), ethylene diamine tetraacetic acid (EDTA), and glycol chitosan (GCS, degree of deacetylation ca. 75.0%, 250 KDa) were purchased from Sigma (St. Louis, MO, USA). Deuterium oxide was provided by Cambridge Isotope Laboratories, Inc. (Cambridge Isotope Laboratories, Tewksbury, MA, USA). These peptides and compounds were used as received without further purification.

### 3.2. Synthesis of Glycol Chitosan-EDTA Conjugate

Glycol chitosan-EDTA was synthesized in a slightly modified way as described by Bernkop-Schnűrch et al. [3,15]. An amount of 500 mg of glycol chitosan was dissolved in 50 mL of deionized double-distilled water. Furthermore, 15 g of EDTA was added to 20 mL deionized double-distilled water and the pH value was kept constant at pH 8 by continuously adding 5 M sodium hydroxide until EDTA was completely dissolved. Deionized double-distilled water was added to make a final volume of 50 mL. Thereafter, the abovementioned solutions were mixed evenly under stirring and the pH value was adjusted to 6.0 with 5 M sodium hydroxide. In order to catalyze the formation of amide bonds between the amino groups of glycol chitosan and the carboxyl groups of EDTA, EDAC was added at a final concentration of 0.1 M. The reaction mixture was incubated at room temperature under continuous stirring for 14 h. The resulting conjugate was isolated by exhaustive dialyzing against 0.05 M sodium hydroxide and then exhaustively against deionized double-distilled water. The purified product was lyophilized and stored at −20 °C until use. Further solubility characterization revealed that glycol chitosan-EDTA was soluble at 10 mg/mL in both pH 6.5 phosphate buffered saline and Hank’s balanced salt solution without Ca^2+^/Mg^2+^ at 25 °C. The resultant solutions are transparent without any precipitate. Moreover, even at pH lower than 2.0, 20 mg/mL glycol chitosan-EDTA is still soluble and the resultant solution is transparent but with increasing viscosity. These results suggest that conjugation of EDTA to glycol chitosan significantly improved the solubility of EDTA.

### 3.3. FTIR and NMR Characterization

Fourier transform infrared (FTIR) spectra measurements were performed using a DTGS detector-equipped Bruker Vertex 80V instrument (Billerica, MA, USA). Data were collected on the transmittance mode over a frequency region of 4000–400 cm^−1^ for 32 interferograms with a resolution of 4 cm^−1^ at 25 °C. Samples were prepared by mixing 200 mg KBr with 1 mg sample. Finally, the spectra were presented in absorption mode after the baseline correction. The ^1^H-NMR spectra measurements were conducted with a Bruker Avance-400 Ultrashield spectrometer (Billerica, MA, USA) equipped with a 5 mm probe at 70 °C [3]. Samples were prepared at 20 mg/mL in deuterium oxide, and pH was adjusted to 10 with 5 M sodium hydroxide (6 µL) prior to running [21]. All chemical shifts were referenced to the HOD peak as a primary reference, and spectral data were collected and analyzed using Bruker’s Topspin software (Version 2.1, Billerica, MA, USA).

### 3.4. Evaluation of Chelating Ability of GCS-EDTA to Calcein

It is well known that calcein fluorescence can be strongly quenched by cobalt ions at physiological pH, while removal of cobalt ion from the complex by EDTA-mediated chelation will lead to a significant increase in fluorescence intensity. Thus, the chelating capacity of the resultant conjugate can be determined by measuring the cobalt ion-chelating-mediated fluorescence recovery of calcein. That is, the fluorescence spectra of calcein coupled with cobalt ions at 1:1 molar ratio were measured with a RF-5301 fluorospectrophotometer (Shimadzu, Tokyo, Japan) in the presence of GCS-EDTA with different concentrations. The excitation wavelength of calcein was set at 490 and its emission was monitored in the range of 500–650 nm. The slits of excitation and emission were set at 5 and 3 nm, respectively. All background effects were subtracted. In addition, the same experiment was done in the presence of EDTA. Finally, the normalized maximum fluorescence emission intensity (calculated as F/F0) of the calcein at each time point was used, where F is the maximum fluorescence emission at each point and F0 is the maximum emission intensity of calcein in the absence of GCS-EDTA or EDTA.

### 3.5. In Vitro Evaluation of Enzyme Inhibition Efficiency of GCS-EDTA

An amount of 0.01 U/mL Leucine aminopeptidase was suspended in 5 mL Krebs phosphate buffer (pH 7.0, 37 °C) which contains 1.3 mM calcium chloride, 1.2 mM magnesium sulfate, 16.5 dibasic sodium phosphate, 120.8 mM sodium chloride, and 4.8 mM potassium chloride. After a 30-min enzymatic activation, the stock solutions of three thymopoietin oligopeptides were added into the above solution and made the final concentration of peptides at 0.1 mM. At specified time intervals after adding peptide, 100 µL aliquots were sampled and diluted with an equal volume of 1 M perchloric acid incubated in an ice bath to stop reaction. Meanwhile, a 100 mL aliquot of the abovementioned buffer was added to the incubation medium. To evaluate the enzyme inhibition efficiency of GCS-EDTA, further investigations were performed with a mixture of the pure enzyme with the conjugates at the same conditions as the degradation experiment. After centrifugation, the concentrations of intact peptide and metabolite in aliquots were analyzed by RP-HPLC as described in our previous studies [3]. All experiments were replicated at least three times.

### 3.6. Calculation of Degradation Clearances

The degradation clearances of three peptides with/without GCS and GCS-EDTA were calculated from the total metabolized peptide amount (ΣM) in the incubation medium at each time point and the area under the peptide concentration-time curve (AUC) according to the equation: ΣM = Cldeg × AUC. Cldeg was determined by linear regression analysis from plots of ΣM versus the AUC at different time points [3,11].

### 3.7. Cytotoxicity Studies

MDCK cells were transferred to 96-well plates (Corning Inc, New York, NY, USA) at a density of 5 × 10^3^ cells/well and cultured for 24 h in a humidified incubator, at 37 °C with 5% CO_2_. The culture medium was then replaced with Dulbecco minimum essential medium containing GCS and GCS-EDTA of different concentrations, respectively. After 24 h coincubation at 37 °C, the medium was replaced with 20 μL of MTT (5 mg/mL in phosphate buffered saline) and 100 μL of incubation medium and incubated for a further 4 h at 37 °C. After incubation, the medium was removed, and 100 μL of dimethyl sulfoxide (DMSO) was added to the residual precipitates. The absorbance of formazan was determined at 490 nm, using an iMark™ microplate reader (Bio-Rad Laboratories, Hercules, CA, USA). Cell viability was expressed as a percentage of the absorbance relative to that of the control. Control cells were not exposed to any materials. The experiments were performed with three replicate wells for each sample and control.

### 3.8. Statistical Analysis

All results are expressed as mean ± standard deviation. Statistical analysis was performed using a two-tailed unpaired Student’s *t*-test in software OriginLab of version 8.5 (OriginLab, Northampton, MA, USA). Difference was considered statistically significant at *p*-values < 0.05.

## 4. Conclusions

In summary, a novel glycol chitosan conjugate was synthesized by forming an amide bond between the amino group of glycol chitosan and carboxyl group of EDTA. The resultant conjugate is soluble in both PBS (pH 6.5) and Hank’s balanced salt solution without Ca^2+^/Mg^2+^ and is compatible with MDCK cells at high concentrations. Moreover, it still keeps the chelation capacity of EDTA to cobalt ions but shows slightly reduced efficiency compared with the free EDTA. Further investigation reveals that GCS-EDTA at low concentrations can significantly protect thymopoietin oligopeptides from leucine aminopeptidase-mediated degradation. This study suggests that GCS-EDTA might be used as an efficient metalloenzyme inhibitor to protect peptides and proteins from enzymatic degradation. Its potential application as a bioadhesive material and permeation enhancer is to be further investigated.
